# Adipocyte Biology from the Perspective of In Vivo Research: Review of Key Transcription Factors

**DOI:** 10.3390/ijms23010322

**Published:** 2021-12-28

**Authors:** Maria N. Evseeva, Maria S. Balashova, Konstantin Y. Kulebyakin, Yury P. Rubtsov

**Affiliations:** 1 Department of Biochemistry and Molecular Medicine, Faculty of Medicine, Lomonosov Moscow State University, 119991 Moscow, Russia; konstantin-kuleb@mail.ru; 2 Laboratory of Bioinformatics Approaches in Combinatorial Chemistry and Biology, Shemyakin-Ovchinnikov Institute of Bioorganic Chemistry of the Russian Academy of Sciences, 117997 Moscow, Russia; 3 Department of Medical Genetics, Sechenov 1st State Medical University, 119991 Moscow, Russia; zimt@list.ru; 4 Laboratory of Molecular Endocrinology, Institute for Regenerative Medicine, Medical Research and Education Centre, Lomonosov Moscow State University, 119991 Moscow, Russia; 5 Laboratory of Molecular Virology, Shemyakin-Ovchinnikov Institute of Bioorganic Chemistry of the Russian Academy of Sciences, 117997 Moscow, Russia; yrubtsov@gmail.com

**Keywords:** transcription factor, obesity, adipogenesis, PPARγ, CREB, CEBP/α, CEBP/β, CEBP/δ, knockout

## Abstract

Obesity and type 2 diabetes are both significant contributors to the contemporary pandemic of non-communicable diseases. Both disorders are interconnected and associated with the disruption of normal homeostasis in adipose tissue. Consequently, exploring adipose tissue differentiation and homeostasis is important for the treatment and prevention of metabolic disorders. The aim of this work is to review the consecutive steps in the postnatal development of adipocytes, with a special emphasis on in vivo studies. We gave particular attention to well-known transcription factors that had been thoroughly described in vitro, and showed that the in vivo research of adipogenic differentiation can lead to surprising findings.

## 1. Introduction

Obesity is a global health threat [1] and the problem is constantly growing [2]. Obesity is characterized as excessive weight gain, as a result of de novo adipocyte differentiation (adipogenesis) or the hypertrophy of existing ones [3]. Adiposity is a constituent of metabolic syndrome and is associated with type 2 diabetes, cardiovascular diseases, and certain types of cancer [4,5,6].

At present, it is known that adipocytes develop from different perivascular subpopulations of mesenchymal stem cells (MSCs), which become committed preadipocytes and, later, adipocytes [7]. Adipose tissue consists of white, beige, and brown adipocytes, which develop [8] and function [9] differently. Since obesity is caused by white adipose tissue (WAT) outgrowth, we will focus on white adipogenesis. Although some insights into beige and brown adipose tissue (BAT) are mentioned, one is recommended to find a profound review of them elsewhere [10,11,12,13,14].

Both the differentiation and maintenance of the differentiated state of adult adipocytes are regulated by transcription factors [15]. Usually, adipocyte differentiation is represented as a cascade of consecutively activated transcription factors [16,17,18,19]. The sequential expression of these factors has been determined in multiple in vitro studies carried out over several decades [20,21,22,23,24,25,26,27,28]. In the current work, we aim to review the roles of the main transcriptional regulators of adipogenesis, as shown in vivo.

## 2. Known Transcriptional Regulators of Adipogenesis in the Light of In Vivo Studies

The process of preadipocyte to adipocyte differentiation is the most thoroughly studied area of adipocyte biology. For decades, it was believed that the transcription factors CREB (cAMP response element-binding protein), CEBPβ and CEBPδ (CCAAT/enhancer-binding protein beta and delta), CEBPα (CCAAT/enhancer-binding protein alpha), and PPARγ (peroxisome proliferator activated receptor gamma) determine the adipogenic differentiation program. A transcriptional differentiation cascade was thought to start with CREB phosphorylation by PKA and ERK1/ERK2 [29] and the consecutive activation of CEBPβ and CEBPδ, which in turn activate CEBPα and PPARγ. Over the years, it was found that this process is much more complicated [30,31,32,33]. We would like to further discuss how the primary in vitro dissection of the differentiation process is specified by in vivo studies.

### 2.1. CREB

CREB is the cAMP response element binding protein, which has three functional isoforms: α, β, and δ. As implied by its name, CREB is a cAMP-dependent transcriptional mediator and a member of the CREB/ATF family. CREB is activated by insulin [29] and used to induce adipogenesis. This finding triggered the investigation of CREB’s role in adipogenic differentiation. Soon, it was found that the stable expression of CREB in 3T3-L1 preadipocytes leads to and is sufficient for adipogenic differentiation. Accordingly, the expression of the CREB dominant negative form leads to adipogenic blocking [34]. An in-depth study of CREB’s function in adipogenic differentiation showed that CREB ablation leads to the loss of PPARγ, C/EBPα, and adiponectin expression. Moreover, the individual ectopic expression of PPARγ, C/EBPα, or C/EBPβ could not rescue the differentiation in CREB-deficient preadipocytes. On the other hand, CREB hyperexpression was able to overcome the adipogenic block induced by CEBPβ downregulation [35,36,37]. Thus, the results of in vitro studies suggest that CREB is a pivotal factor for adipocyte differentiation and acts in several stages of adipogenesis.

Several CREB knockout mouse models have been generated. If α and δ isoforms are deactivated, CREBβ isoform hyperexpression takes place; thereby, it was found that these αδ-deficient mice had mostly intact phenotypes, except for long-term memory deficiency [38]. On the contrary, it was found that CREB null mice (α, β, and δ were deactivated) die immediately after birth. These mice are smaller than their wild-type littermates, but no abnormalities in the adipose tissue depots have been reported [39]. It is worth noting that CREB null mice demonstrate no developmental defects in their adipocyte tissue; thus, other mechanisms must compensate for CREB deficiency. Since CREB knockout in embryos is a lethal mutation, Lee D. downregulated CREB only in adipose tissue [40]. For this purpose, Creb-loxP mice [41,42] were crossed with Adipoq-Cre mice [41,43]. The combination of these alleles leads to Creb gene knockout in cells with an active adiponectin promoter. The body weights and lean-to-fat mass ratios of 3 to 5 month old mice with CREB selectively inactivated in their adipose tissue were found to be comparable to those of their wild-type littermates. The histological pattern of adipose tissue was not affected by CREB knockout, coinciding with the normal transcriptome of Creb−/−adipocytes. The only substantial discrepancy found was a moderate decrease in fasting-induced lipolysis in knockout animals [40]. However, it should be noticed that in vitro studies have shown that CREB functions upstream of adiponectin [34]. This is why CREB downregulation after the activation of adiponectin promoter can come too late to evaluate CREB function in adipogenesis in vivo. As shown earlier, in vitro CREB activates C/EBPβ to further induce adipogenic differentiation [36]. However, according to the in vivo study of Lee at al. [40], the role of CREBs in white adipogenesis can be substituted by other CREBs/ATFs [37], or by transcription factors unrelated to CREB.

### 2.2. C/EBPβ and C/CEBPδ

C/EBPs is a family of transcription factors containing six members (C/EBPα, β, γ, δ, ε, and ζ, or CHOP) with a basic-leucine zipper domain [44]. In vitro research has shown that C/EBPβ stimulates adipogenesis while its downregulation blocks it [31]. Later, the mechanism of C/EBPβ action was recognized—that is, the transcriptional activation of PPARγ 2 and CEBPα [45]. However, the results obtained in vivo call into question the data of in vitro experiments.

In the profound research of Tanaka et al., C/EBPβ (−/−), C/EBPδ (−/−), and C/EBPβ (−/−)·δ (−/−) phenotypes were analyzed [46]. Specifically, it was found that 35% and 85% of C/EBPβ (−/−) and C/EBPβ (−/−)·δ (−/−) mice die soon after birth; thus, it seems that C/EBPβ and C/EBPδ are interchangeable to some degree.

C/EBPβ (−/−)·δ (−/−) mice have a reduced epididymal WAT and BAT. The BAT of C/EBPβ (−/−) mice is significantly and C/EBPδ (−/−) mice is slightly reduced in lipid content and UCP1 expression, while the WAT of both these transgenic models is comparable to control. Surprisingly, in the BAT of all transgenic models, PPARγ and C/EBPα were expressed normally; however, the downregulation of UCP1 mRNA from substantial (in C/EBPβ (−/−)·δ (−/−) mice) to slight (in C/EBPδ (−/−) mice) was observed. The fact that PPARγ and C/EBPα can be properly expressed despite C/EBPβ (−/−)·δ (−/−) knockout contradicts the results of previous in vitro studies. Even more interesting is the fact that, despite the normal expression of PPARγ and C/EBPα, impaired differentiation in BAT is observed. Therefore, in the interscapular BAT, C/EBPα and PPARγ expression is independent of C/EBPβ (−/−) and/or C/EBPδ (−/−), and can be induced by other means.

MEFs (mouse embryonic fibroblasts) from the knockout animals were further differentiated into adipocytes with the standard protocol. MEFs from C/EBPβ (−/−) mice exhibited significantly reduced differentiation (immature adipocytes) compared with wild-type cells, while the differentiation of C/EBPδ (−/−) MEFs was reduced only slightly (mature adipocytes). By contrast, MEFs from double knockout animals showed nearly no differentiation. It was shown that C/EBPα expression deteriorates only in double-knockout mice, while PPARγ expression is reduced markedly in both C/EBPβ (−/−) and C/EBPβ (−/−)·δ (−/−) fibroblasts.

The researchers have analyzed three different types of cells—BAT, epididymal WAT, and MEF—and concluded that C/EBPβ (−/−)·δ (−/−) knockout had different effects on the adipogenic programs of these cells.

This in-depth study demonstrated that the transcriptional networks governing adipocyte biology are site- (WAT, BAT, and MEFs) and time- (embryonic vs. adult) specific. The shortcoming of this work is, however, the fact that C/EBPβ and δ genes were knocked out nonselectively, thus meaning that metabolic changes induced by whole-body knockout can influence the results. It would therefore be interesting to compare the results obtained with selective models with C/EBPβ and δ gene knockout in adipose tissue.

**Figure 1:**
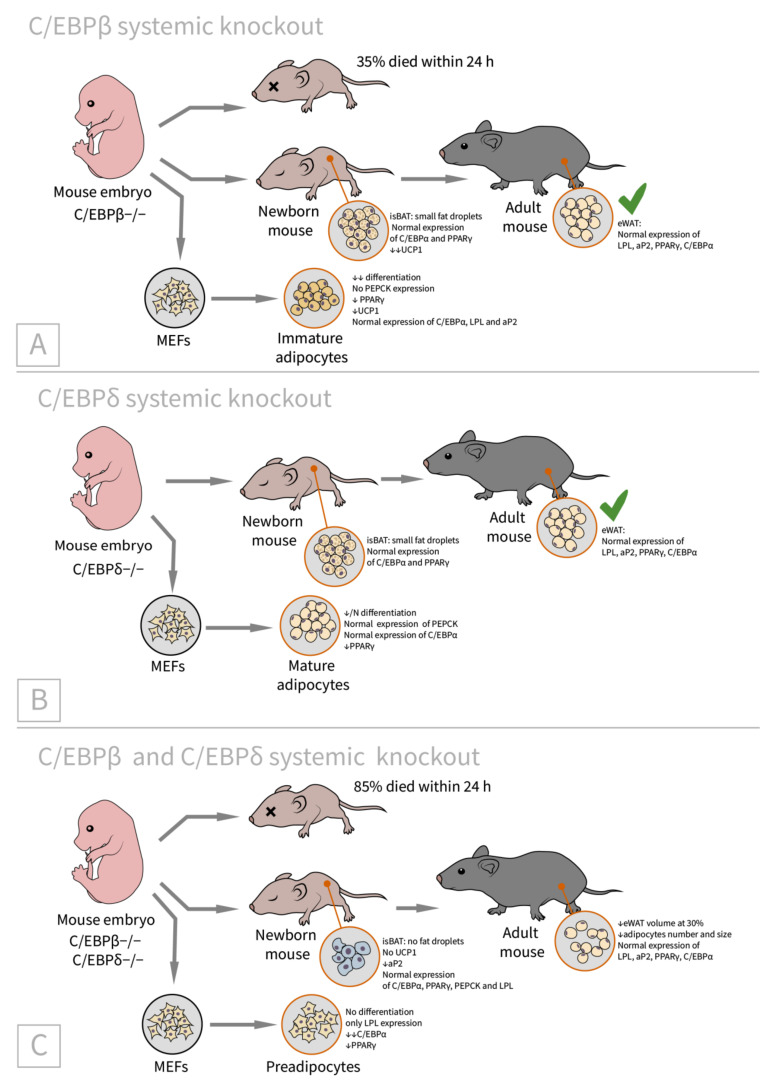
An illustration of the phenotypes of systemic C/EBPβ (−/−), C/EBPδ (−/−), and C/EBPβ (−/−)∙C/EBPδ (−/−) transgenic mice. (**A**) C/EBPβ knockout: 35% of newborn C/EBPβ (−/−) mice died within 24 h after birth. The surviving mice had small lipid droplets in isBAT (interscapular BAT) with a normal expression of C/EBPα and PPARγ. Adult mice had normal eWAT (epididymal WAT) and a decreased expression of UCP1 (the functional marker of terminally differentiated BAT). The differentiation of mouse embryonic fibroblasts (MEFs) from C/EBPβ (−/−) mice was significantly impaired. (**B**) C/EBPδ knockout: small lipid droplets in isBAT, normal eWAT in adults, and MEF differentiation were normal or slightly impaired. (**C**) C/EBPβ + C/EBPδ knockout: 85% of newborn C/EBPβ (−/−)∙C/EBPδ (−/−) mice died within 24 h after birth. The surviving mice had no lipid droplets in isBAT, and UCP1 expression was markedly reduced, with a normal expression of C/EBPα and PPARγ. Adult mice had a reduced volume of eWAT with a normal expression of C/EBPα and PPARγ. MEFs did not differentiate into mature adipocytes.

An illustration of the described phenotypes of C/EBPβ (−/−), C/EBPδ (−/−), and C/EBPβ (−/−)∙C/EBPδ (−/−) transgenic mice can be found in Figure 1.

### 2.3. C/EBPα

The critical role of C/EBPα in adipogenic differentiation in vitro has been extensively demonstrated, previously [22,47,48,49,50,51,52,53,54]. Thus far, several mouse models aiming to elucidate the role of C/EBPα in adipose tissue in vivo have been used. In this section, we will discuss some of them further.

First attempt to knockout C/EBPα in a mouse led to a newborn lethality within 8 h after birth due to hypoglycemia induced by disrupted gluconeogenesis in the liver [55]. These animals encounter various severe defects apart from defective gluconeogenesis [55,56,57,58,59]. Linhart et al. faced the problem of perinatal lethality by overexpressing C/EBPα exclusively in the liver of C/EBPα-deficient mice [60]. This approach improved the survival rates of the transgenic animals by 3 times. The adipose tissue depots of 7-day old mice were analyzed: WAT was almost completely absent, while BAT and breast fat pads, which consisted of WAT, were normal. These C/EBPα-deficient mice showed fatty liver, postprandial hyperlipidemia, hyperinsulinemia, while glucose levels were normal. Thus it is apparent from this study that different adipose depots have an uneven dependency on C/EBPα.

Somewhat common results were obtained by Yang et al. [61]. In this work, the role of C/EBPα in the postnatal period was assessed by the inducible knockout of C/EBPα with poly(I:C) administration. In these transgenic mice, C/EBPα was effectively knocked out in the liver, spleen, WAT, BAT, pancreas, lung, and kidney. Namely, C/EBPα downregulation in both newborn and adult mice was accompanied by biphasic changes: for the first 2 weeks, the transgenic animals were phenotypically indistinguishable from control animals. However, on the 16th day after poly(I:C) administration, severe growth retardation and weight loss were seen. All animals died within a month after C/EBPα-inducible knockout. Specifically, the WAT was substantially reduced in size while the BAT was enlarged. Moreover, transgenic animals developed hepatic steatosis, there was a loss of triglyceride in WAT but not in BAT, and the animals were hypoglycemic and hypoinsulinemic.

**Figure 2:**
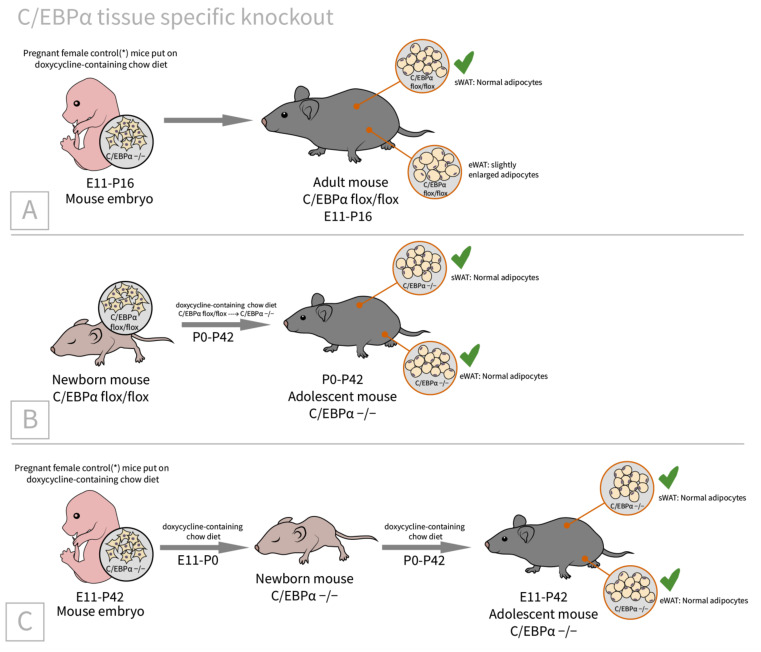
Embryonic and neonatal adipose tissue development was C/EBPα-independent. (**A**) Pregnant female control mice (*mice contain only Adn-rtTA and C/EBPα flox/flox) were given doxycycline-supplemented chow during the E11-E18 embryonic days (the period of subcutaneous WAT development), which induced the C/EBPα knockout in the embryos. After E18 doxycycline supplementation was stopped, C/EBPα expression was restored. (**B**) Neonatal C/EBPα flox/flox pups were put on doxycycline-supplemented chow from P0 (postnatal day 0), which induced C/EBPα knockout, until P42 (the period of epididymal WAT development). Both epididymal and subcutaneous WAT were comparable to the control. (**C**) Pregnant female control mice were given doxycycline-supplemented chow from E From P0 until P42, newborn mice continued to receive doxycycline-supplemented chow. Thus, C/EBPα was knocked out during both critical periods of WAT development (embryonic period, critical for subcutaneous WAT, and postnatal period, for epididymal WAT). Either subcutaneous or epididymal WAT were comparable to the control.

A more sophisticated approach was provided by Wang et al. The authors introduced a tissue-specific deletion of C/EBPα [62], where the transgenic animals carried floxed C/EBPα, Cre-recombinase under tetracycline response element (TRE), and reverse tetracycline-controlled transactivator (rtTA) under adiponectin promoter; thus, doxycycline administration induced selective knockout of floxed C/EBPα in the adipose tissue. This model revealed several surprising results. For instance, it was found that C/EBPα knockout in the embryonic period (E14-18) has no influence on either subcutaneous or epididymal WAT development, and these tissues seem to be C/EBPα-independent (Figure 2). Conversely, PPARγ knockout in the same period leads to the complete absence of subcutaneous WAT and the compensatory overgrowth of epididymal WAT (epididymal WAT develops postnatally, which is why PPARγ knockout in the embryonic period had no influence on epididymal WAT development).

**Figure 3:**
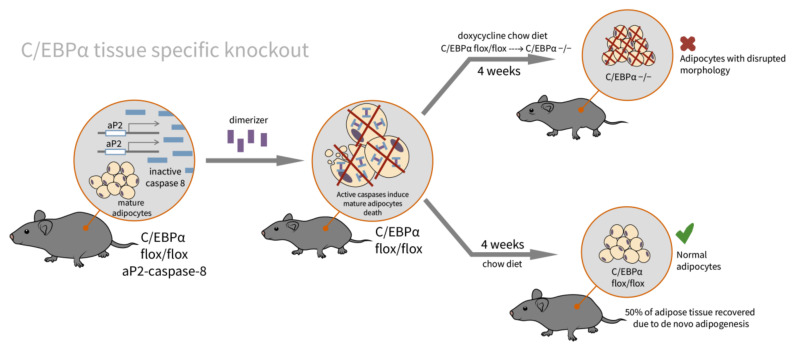
C/EBPα is indispensable for de novo adipogenesis in adults. The transgenic line was derived by crossing the inducible C/EBPα floxed/floxed mice with FAT-ATTAC mice (FAT apoptosis through triggered activation of caspase-8). FAT-ATTAC mice expressed an inactive form of caspase-8 under the aP2 promoter (thus, in adult adipocytes). A single treatment with a dimerizer activates caspase-8 and induces apoptosis in mature adipocytes. A week after the dimerizer treatment, the fat depots were significantly reduced. These mice were then put on a doxycycline chow diet (for C/EBPα knockout) or on a chow diet (C/EBPα was expressed). In mice on the chow diet, their fat depots recovered to approximately 50% of the original tissue, while in mice on the doxycycline chow diet, their fat pads were still reduced.

In addition to embryonic development, the role of C/EBPα was investigated in adult adipogenesis. In the experiments with caspase-induced apoptosis in mature adipocytes, the role of C/EBPα in de novo adipogenesis was evaluated and it was shown that de novo adipogenesis is C/EBPα-dependent (see Figure 3).

**Figure 4:**
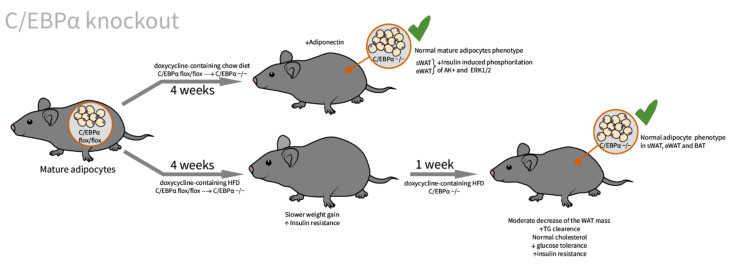
Metabolic changes in mice with a C/EBPα knockout in adipose tissue. Four weeks under normal chow feeding led to a normal phenotype of mature adipocytes, but impaired the insulin-stimulated phosphorylation of Akt and ERK1/2 in WAT depots and a decrease in the systemic adiponectin level. Four weeks under HFD conditions induced a slower weight gain (compared to C/EBPα-expressing mice), which is thought to be due to adipocyte hypertrophy. After another one to two weeks of HFD feeding, C/EBPα −/− mice begin to lose weight moderately (presumably due to the termination of hypertrophic adipogenesis), while the normal adipocyte morphology in WAT and BAT depots was retained.

However, under the HFD conditions, C/EBPα knockout mice continued to slowly gain weight until weeks 4–5, when the first signs of weight decrease were seen. It seems that the weight gain despite the C/EBPα knockout can be attributed to the adipocyte hypertrophy, rather than de novo adipogenesis (Figure 4). These two experiments provide evidence that C/EBPα is indispensable for adult de novo adipogenesis, but not important in HFD-induced adipocyte hypertrophy or in adipocyte morphology maintenance, since C/EBPα knockout in mature adipocytes has no influence on the cell number and morphology. However, significant impairments in the insulin-stimulated phosphorylation of Akt and Erk1/2 in WAT and a reduction in the circulating adiponectin level were seen.

C/EBPα is indispensable for adipocyte expansion in ob/ob mice. By contrast, beige adipogenesis in adult mice is fully C/EBPα-independent [62].

Thus, the results of C/EBPα knockout in adipose tissue, as presented by Yang [61], are in agreement to some degree with those of Linhart [60]: C/EBPα is important in the differentiation and maintenance of WAT but not non-redundant in BAT. Both studies point to the development of fatty liver in transgenic animals, but some differences are apparent as well; in one study, animals were hyperinsulinemic with a normal glucose level, while Yang et al. reported opposing results: the mice had hypoglycemia and hypoinsulinemia. While Wang et al. generated a more specific knockout in adipose tissue and the study demonstrated opposing results: WAT develops independently of C/EBPα. Despite the tissue-specific inducible approach used by Wang et al., C/EBPα is knocked out only after adiponectin promoter activation—a shortcoming that permits a confident evaluation of only the results obtained in mature adipocytes. Once again, it is noted that the role of C/EBPα in adipogenesis differs according to the cell type and stage of development.

It seems that future work will be needed to more specifically evaluate the described phenotypes and further elucidate the role of C/EBPα in the tissues in which it is expressed.

### 2.4. PPARγ

The essential transcription factor governing adipocyte biology is thought to be PPARγ [63,64]. Detailed information about PPARγ can be found in several excellent reviews [65,66]. In the present paper, we focus on the functions of PPARγ in adipose tissue in vivo.

Initial attempts to study PPARγ function in vivo have led to embryonic lethality due to placental dysfunction [67,68]. Several rescue studies with global PPARγ deletion were conducted [68,69,70,71]. Since systemic PPARγ deletion strongly influences whole-body homeostasis, a more specific approach was needed to study knockout effects in adipose tissue.

Thus, for the tissue-specific deletion of PPARγ, Cre-recombinase under aP2 promoter was introduced [72,73,74,75], with some inconsistency being shown between these studies [73,75]. For instance, in the study of He et al. under HFD conditions, fat-specific PPARγ knockout mice were found to be hyperinsulinemic and to display insulin resistance (IR) in fat and liver, but not in muscle [73], while in another study by Jones et al., PPARγ-selective knockout under HFD challenge leads to muscle insulin resistance but an overall improvement in insulin sensitivity due to the increased glucose uptake in the liver [75]. The use of aP2-promoter has an obstacle: aP2 is a late marker of adipogenesis and is normally expressed after PPARγ and C/EBPα activation [48,63]. Thus, attempts were made to generate tamoxifen-inducible PPARγ knockout under aP2 promoter [72]; however, the aP2 gene is not tissue-specific. To overcome this difficulty, a more fat-specific Cre-mediated transgenic line was generated with the help of an adiponectin promoter [76]. Since the adiponectin promoter is activated at the late stage of adipogenesis [77], the knockout is thought to happen in nearly differentiated adipocytes that already express adiponectin. The phenotypic changes in these transgenic mice were evaluated during their 3rd month after birth and included adipose tissue deficiency in all adipose depots, fatty liver, enlarged pancreatic islets, abnormal mammary gland development, and a substantial decrease in the plasma levels of leptin, resistin, and adiponectin, accompanied by insulin resistance.

Induction of PPARγ knockout at the late stages of adipogenesis, several inconsistencies between fat mass and insulin resistance in different organs, and the timing of the appearance of lipodystrophy in PPARγ phenotypes (a few days in one study [72] and several months in another [73]), as well as the striking phenotype of Adipo-Cre PPARγ −/− mice [76], due to the pleiotropic function of PPARγ, prompted researchers to generate a new transgenic model with inducible PPARγ knockout.

**Figure 5:**
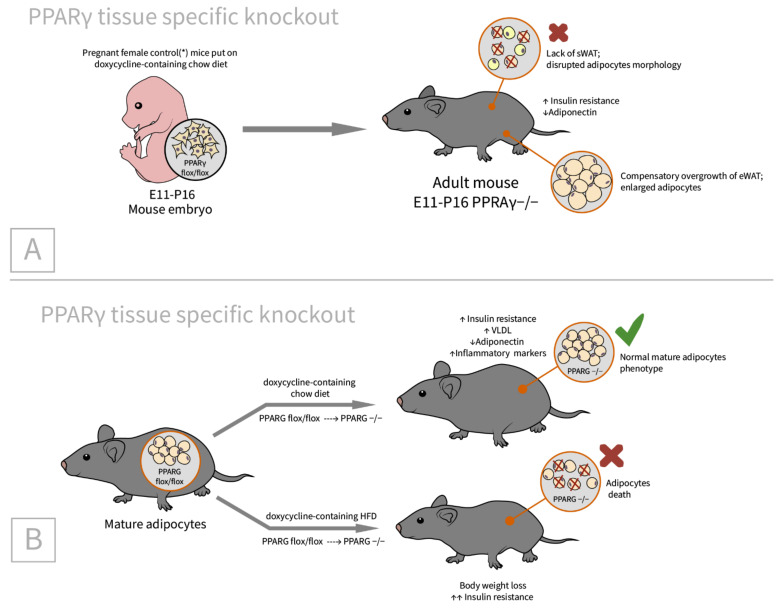
PPARγ is essential in embryonic adipose tissue development and adult adipogenesis. (**A**) Pregnant female control mice (*mice contain only Adn-rtTA and C/EBPαflox/flox) were given doxycycline-supplemented chow from day E11 until birth. Newborn pups were given doxycycline-supplemented chow from P0 until PThus, PPARγ was knocked out in adipose tissue from E11 until PThese mice had miniscule subcutaneous WAT and increased epididymal WAT (due to compensatory overgrowth). (**B**) Under normal chow diet conditions, the adipose tissue of adult mice with the PPARγ knockout had normal morphology, though these mice had serious metabolic abnormalities: insulin resistance, increased VLDL (very low-density lipoprotein), and decreased adiponectin levels. In contrast, PPARγ −/− adult mice on a doxycycline-containing a high-fat diet developed severe weight loss and insulin resistance.

As mentioned above, to study the role of C/EBPα in adipose tissue biology [62], PPARγ conditional knockout mice were used as a control. Thus, in parallel with C/EBPα, the authors investigated the role of PPARγ in embryonic adipogenesis and showed that, in contrast to C/EBPα, PPARγ is indispensable for the embryonic differentiation of WAT [62]. For the further study of the role of PPARγ in mature adipocytes, Wang et al. used the transgenic model, for which PPARγ was conditionally knocked out by doxycycline administration (Figure 5) [78].

The authors showed that PPARγ knockout in mature adipocytes that lasted for two weeks, had no substantial influence on adipocyte biology in vivo or in vitro (Figure 5B). However, a systemic reduction in insulin sensitivity, as shown by the substantial decrease in insulin-induced Akt and Erk1/2 phosphorylation in epididymal WAT, was found. A reduction in insulin sensitivity was also observed in the liver, alongside a profound reduction in systemic adiponectin levels. At the same time, prolonged PPARγ deficiency for more than a month, exacerbated these changes and altered the adipogenic transcription factors LXRα, SREBP1c, and C/EBPα in subcutaneous WAT.

**Figure 6:**
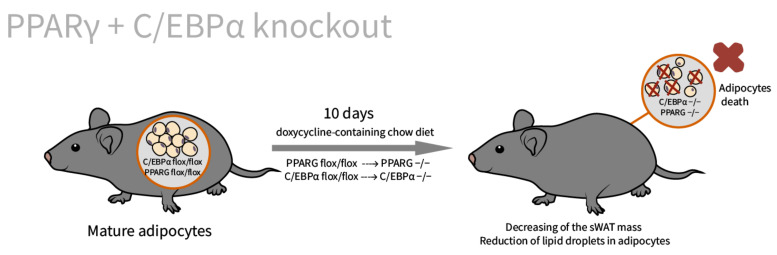
Overlapping functions of PPARγ and C/EBPα in maintaining the survival of mature adipocytes. The double knockout of PPARγ and C/EBPα was induced by doxycycline-containing chow diet supplementation for 10 days. The weight of the transgenic mice has not changed, while almost all adipocytes of subcutaneous WAT were disrupted and dead. Epididymal WAT size was comparable to control.

As shown previously [62], C/EBPα and PPARγ have different transcription targets in vivo. However, the double knockout of C/EBPα and PPARγ in vitro and in vivo leads to rapid adipocyte disruption and the death of subcutaneous WAT (Figure 6). Surprisingly, epididymal WAT size of PPARγ (−/−)∙C/EBPα (−/−) mice is comparable to control; thus, it seems that PPARγ and C/EBPα have overlapping functions in maintaining the survival of mature adipocytes.

## 3. Discussion and Conclusions

### 3.1. Transcription Factor Summary

For this study, we reviewed the transcriptional axis of adipogenesis in the light of in vivo studies. To the best of our knowledge, no tissue-specific knockout of C/EBPβ has been recorded to date. In the study of Tanaka et al. [46], three systemic knockout phenotypes (C/EBPβ, C/EBPδ, and C/EBPβ·C/EBPδ) were evaluated. However, due to substantial neonatal lethality in C/EBPβ (−/−)·δ (−/−) (85%) and C/EBPβ (−/−) (35%) mice, their phenotypes can be underestimated. One of the interesting outcomes of this study is the fact that C/EBPα and PPARγ can be induced apart from C/EBPβ and C/EBPδ expression, but despite the expression of these “master regulators”, epididymal WAT is significantly reduced in size. This shows that, at least in the case of systemic knockout, C/EBPβ and C/EBPδ transcription factors are indispensable in epididymal adipose tissue. However, it would still be interesting to confirm these results in tissue-specific models.

The role of C/EBPα in adipocyte biology has been investigated more extensively. The systemic knockout of C/EBPα [60,61] and inducible tissue-specific knockout [62] lead to different outcomes: the first two studies stressed the essential importance of C/EBPα in adipose tissue, while inducible tissue-specific knockout research demonstrated that C/EBPα is indispensable for adipocyte expansion under metabolic challenges and in de novo adipogenesis. By contrast, late embryonic and neonatal embryogenesis, mature adipocyte state maintenance, and hypertrophy are C/EBPα-independent.

It was anticipated that PPARγ and C/EBPα would share a common transcriptome [79]; however, in vivo research has discovered that their transcriptional programs are quite different [62], except for mature adipocyte state maintenance, for which both transcription factors act synergistically, so that deleting PPARγ and C/EBPα simultaneously would lead to adipocyte death [78]. PPARγ function in adipocyte biology is pivotal in nearly all circumstances, as has been conformed in several studies [62,73,75,76] with the exception of only short-term knockout in mature adipocytes [78].

Apparently, several discussed transcription factors are interchangeable in some way or function alongside dozens of others, whether known [26,80], new [81,82,83], or not yet identified. The phenotypic diversity of the reviewed models, even in the case of the same gene knockout, demonstrates that there remains a lot to learn about adipogenesis. Since the exact lineage markers of preadipocyte cells are still under investigation, data on the roles of the discussed transcription factors in embryonic development and early postnatal period are still lacking.

It should be stressed that the regulation of metabolic processes differs between humans and mice [84]. Hence, the deciphering of genetic polymorphism using GWAS (genome-wide association studies) can be considered as a good starting point from which to find new molecular regulators of human metabolism [85]. There is no doubt that the candidate regulators identified by GWAS should be thoroughly tested, to prove their involvement in metabolic processes. For GWAS-discovered genes, such as FTO [86], FAM13A [87], CDKAL1 [88], Hhex [89,90], and others, work has already begun.

A summary of the described transgenic models can be found in Table 1.

### 3.2. The Role of the Transcription Factor Imbalance in the Development of Metabolic Disorders

Obesity and metabolic disorders, such as type 2 diabetes, are tightly interconnected [91]. However, the precise mechanism underlying this association is still under investigation. It is known that the excessive weight gain can be due to adipocyte hypertrophy or hyperplasia [92]. It has been noticed that people with hyperplastic obesity, which is characterized by active de novo adipogenesis, are metabolically healthy [93,94,95,96], while hypertrophic overweight is associated with type 2 diabetes [97,98,99].

In view of these facts, it was proposed that the fat overfilling of existing adipocytes leads to trafficking incoming triglycerides to other tissues (for instance, the liver and the skeletal muscle) [100,101], where they enter the Randle cycle, competing with glucose and inducing insulin resistance [102,103]. Human lipodystrophies, both congenital, meaning they are caused by genetic defects in the development and/or differentiation of adipose tissue (for example, mutations in PPARG) [104,105], and acquired as an adverse effect of some medications (HIV-1 protease inhibitors), can be viewed as an example supporting this notion: body fat absence induces insulin resistance, hypertriglyceridemia, and hepatic steatosis [106]. Thus, the failure of preadipocytes to proliferate and differentiate [107] is thought to be at the core of metabolic disorders [108,109]. For instance, when hyperplastic (protective) obesity is induced by injecting an adipogenic cocktail into mouse subcutaneous adipose tissue, this results in an improvement in glucose tolerance and insulin sensitivity [110]. Since transcription factors govern adipocyte proliferation and differentiation, the dysfunctions in transcriptional regulation seen in obesity [111,112] can be a clue to the mechanism of obesity-associated metabolic disorders.

As we have mentioned previously, de novo adipogenesis in adult mice WAT is C/EBPα- and PPARγ-dependent. Additionally, the disruption of adipose tissue under HFD conditions in C/EBPα −/− and PPARγ −/− and in ob/ob C/EBPα −/− mice induces metabolic changes resembling those in people with PPARγ deficiency [113]. In humans, the dominant negative mutations in PPARγ are associated with severe insulin resistance and type 2 diabetes [114], as well as nine newly identified loss-of-function mutations in this gene [115]. The administration of thiazolidinediones (PPARG agonists) substantially improves IR in mice [116] and humans [117]. However, these effective drugs cause serious adverse events due to their systemic action, including edema, bone fractures, and the exacerbation of preexisting heart failure [118].

Accordingly, interventions other than thiazolidinediones, can be used to correct metabolic disorders associated with obesity. Non-coding RNAs should be considered as potential modulators of transcriptional activity in adipocytes, and, as such, these small FDA-approved drugs are potentially promising tools for treating metabolic disorders [119]. The mechanisms involved in the regulation of adipogenic differentiation by certain miRs remain unclear [120]. However, miRs that target principal regulators of adipogenesis, such as PPARγ (MiR-27) [121] and C/EBPα (MiR-31), have been identified [122]. These known repressors of adipogenesis are being actively investigated as possible targets for the treatment of obesity.

Adipocyte transcriptional activity is influenced by lifestyle factors, such as physical exercise. Early work in this field showed that exercise can upregulate the production of endogenous ligands of PPARγ, thereby increasing its transcriptional activity [123], while recent studies have shown that physical activity is associated with a direct increase in PPARγ expression [124]. The same is true for CEBP isoforms, as a strong negative correlation has been reported between exercise intensity and the expression of CEBPA and CEBPB in the obese state [125].

Transcription factor expression and activity in adipocytes are also modulated by food intake and dietary factors. Specifically, several food ingredients affect PPARG expression and obesity-related parameters. Among them are anthocyanins [126], n-3 polyunsaturated fatty acids [127,128,129], olive leaf extract [130], geranylgeraniol (GGOH) [131], rice bran [132], and other compounds.

From the studies in mice reviewed above, one can see that transcription factors act in a depot-specific manner—for instance, C/EBPβ knockout in BAT induces a differentiation block while WAT cells display a normal morphology, and there is a substantial heterogeneity in the roles of transcription factors between MEF and mature adipocytes. It is also known that inguinal WAT expands due to hypertrophy, while epididymal WAT in the same mice is prompted to de novo adipogenesis [133]. The depot specificity also occurs in humans, as PPARG agonists not only improve IR, but also induce protective adipogenesis in femoral subcutaneous adipose tissue and reduce visceral obesity (which is metabolically unhealthy), acting in a depot-specific manner [117].

Thus, one can see that the transcriptional regulation of adipogenesis is not a straightforward uniform cascade of sequentially activating transcription factors, as was thought earlier, but rather a cell type-, depot-, and developmental stage-specific transcriptional network, whose pattern is only recently starting to became clear. Further studies of the transcriptional regulation in adipocyte biology should be performed with the usage of appropriate mouse models. The existing two research methods for studying adipocyte biology (lineage tracing and tissue-specific transgenic models) should be combined to answer the following questions: what are preadipocyte cells? Where (in what fat depot and in what histological surrounding) are they located? Additionally, when (under what circumstances) do they act to exert their function? Answers to these questions can help to develop novel and effective cell- and tissue-specific medical interventions with fewer side effects.

## Figures and Tables

**Table 1:** A summary of tissue-selective and systemic transgenic models, describing functions of CREB, C/EBPβ, C/EBPδ, C/EBPα, and PPARγ transcription factors in adipose tissue in vivo.

Transcription Factor Knockout/Transgenic Model/Developmental Stage Analyzed	Adipose Tissue Phenotype	Systemic Phenotype	Comments
CREB [40]/C57BL/6J background, AT-specific CREB(−/−)/adults 3–5 months	Lean-to-fat mass ratio and body weights were comparable with control, moderate decrease in fasting-induced lipolysis	Non-esterified fatty acids (NEFA) levels were 40% lower compared to control	CREB knockout under adiponectin promotor—may be too late to evaluate CREB function
CEBP β [46]/C57BL/6 background, systemic C/EBPβ(−/−)/newborns (BAT) adults (WAT)	eWAT: LPL, aP2, PPARγ, and C/EBPα are expressed comparably to control isBAT: Lipid accumulation is only slightly impaired, UCP1 expression is reduced MEFs: differentiation is significantly reduced, cells expressed LPL and aP2 but not PEPCK (immature adipocytes), C/EBPα expression is comparable with WT, and PPARγ is reduced substantially	35% of knockout animals die within early neonatal period	isBAT: cells with small lipid droplets suggested differentiation block is at the immature adipocyte stage
CEBP δ [46]/C57BL/6 background, systemic C/EBPδ(−/−)/newborns (BAT) adults (WAT)	eWAT: LPL, aP2, PPARγ, and C/EBPα are expressed comparably to WT isBAT: Lipid accumulation is only slightly impaired, UCP1 expression is slightly reduced MEFs: differentiation is slightly reduced, cells express PEPCK (mature adipocytes), C/EBPα expression is comparable with WT, and PPARγ is reduced slightly	N/A	isBAT: the adipocytes are the same as the wild-type or slightly reduced in size
CEBP β + δ [46]/C57BL/6 background, systemic C/EBPβ(−/−)C/EBPδ(−/−)/ newborns (BAT), adults (WAT)	eWAT (adult): 30% lower than in control mice, LPL, aP2, PPARγ, and C/EBPα are expressed comparably to WT isBAT (newborn): no fat droplets, UCP1 mRNA almost absent, aP2 mRNA is reduced by half, PEPCK and LPL are expressed normally MEFs: only LPL expression (preadipocytes stage), C/EBPα is reduced markedly, and PPARγ is reduced substantially	No histological abnorm- alities in the liver and lung, no hypoglycemia	All mice develop system growth defects, 85% of double knockout animals die within 24 h, and the remaining phenotypes were analyzed (thus the phenotype may be underestimated)
C/EBPα [60]/FvB/N mice, systemic C/EBPα knockout except for the liver/newborn and 7-days old mice	WAT: absent except for mammary fat pad (morphologically similar to control) sBAT: present, enlarged, and contains larger fat vacuoles. UCP mRNA is reduced in newborns; by 7 days of age mRNA levels of UCP, FAT, LPL, and aP2 are comparable with control	Agranulocytosis and pulmonary dysplasia in newborns Postprandial hyperlipidemia, fatty liver, and 60% reduction in serum leptin levels	N/A
C/EBPα [61]/mixed background C57BL6, SVE129, and CBA strains/C/EBPα systemic inducible knockout/newborns and 3 months old adults	WAT: decreased in size, triglyceride loss BAT: either larger than or similar to control	fatty liver, hypoglycemia, hypocholesterolemia, hypoinsulinemia, hyperammonemia, and hyperproteinemia	All animals display biphasic changes in phenotype: the first 2 weeks phenotype of transgenic animals are comparable with control, the subsequent 2 weeks is accompanied by severe weight loss, hypophagia and death.
C/EBPα [62]/C57BL/6J background/C/EBPα tissue specific doxycycline inducible knockout/embryos, newborns, adults, and MEFs	WAT: adults with embryonically knocked out C/EBPα have comparable tissue mass and normal adipocyte size and morphology; insulin-stimulated phosphorylation of Akt and Erk1/2 is significantly impaired BAT: slightly enlarged adipocytes	Decrease in adiponectin to 14% of control; impaired glucose tolerance, insulin resistant on HFD	indispensable for adipocyte regeneration in adults, and expansion under HFD conditions, not essential in terminal embryonic adipogenesis, and mature adipocyte survival
PPARγ [62]/C57BL/6J background/PPARγ tissue specific doxycycline inducible knockout/embryos, newborns, adults, and MEFs	sWAT: small size, disrupted adipocyte morphology in Adn-PPARγ−/−(E11-P16) * male offspring eWAT: compensatory increased (by 36%)	Adiponectin reduction by 24% in Adn-PPARγ−/−(E11-P16) mice. Insulin resistance in adipose tissue and liver (adults) [78]	Indispensable in nearly all circumstances except for short-term knockout in adult mature adipocytes in vivo [78]

Abbreviations used in the Table: AT (adipose tissue); BAT (brown adipose tissue); sBAT (supraclavicular brown adipose tissue), eWAT (epididymal white adipose tissue); sWAT (subcutaneous white adipose tissue); NEFA (non-esterified fatty acids); HFD (high fat diet); * Adn-PPARγ−/−(E11-P16)—animals with PPARγ inducible deletion from embryonic day E11 until postnatal day P16; N/A—not applicable.

## Data Availability

Not applicable.
